# Pravastatin promotes type 2 diabetes vascular calcification through activating intestinal *Bacteroides fragilis* to induce macrophage M1 polarization

**DOI:** 10.1111/1753-0407.13514

**Published:** 2023-12-19

**Authors:** Cong Chen, Zheng‐Feng Liang, Yu‐Qi He, An‐Qi Li, Yan Gao, Qun‐Wen Pan, Jing‐Song Cao

**Affiliations:** ^1^ The First Affiliated Hospital, Institute of Endocrinology and metabolism, Center for Clinical Research in Diabetes, Hengyang Medical School University of South China Hengyang China; ^2^ The First Affiliated Hospital, Department of Laboratory Medicine, Hengyang Medical School University of South China Hengyang China; ^3^ Guangdong Key Laboratory of Age‐Related Cardiac and Cerebral Diseases, Institute of Neurology, Affiliated Hospital of Guangdong Medical University Zhanjiang China

**Keywords:** *Bacteroides fragilis*, macrophage, pravastatin, type 2 diabetes, vascular calcification

## Abstract

**Background:**

Pravastatin is an oral lipid‐lowering drug, commonly used by patients with diabetes that is positively correlated with the occurrence of vascular calcification (VC), but the mechanism is unclear.

**Methods:**

In this study, 16S rRNA sequencing and qRT‐PCR wereused to detect the differential gut bacteria. Metabolomics and ELISA were used to analyze the differential metabolites. qRT‐PCR and western blotting (WB) were used to detect genes expression. Flow cytometry was used to analyze macrophage phenotype. Immunohistochemistry was used to analyze aortic calcification.

**Results:**

We found that gut *Bacteroides fragilis* (*BF*) increased significantly in patients who took pravastatin or type 2 diabetes (T2D) mice treated with pravastatin. In vitro experiments showed that pravastatin had little effect on *BF* but significantly promoted *BF* proliferation in vivo. Further analysis showed that *ArsR* was an important gene for pravastatin to regulate the activation of *BF*, and overexpression of *ArsR* significantly promoted the secretion of 3,4,5‐trimethoxycinnamic acid (TMCA). Importantly, pravastatin significantly promoted *BF* secretion of TMCA and significantly increased TMCA secretion in T2D patients or T2D mice. TMCA had little effect on vascular smooth muscle cell calcification but significantly promoted macrophage M1 polarization, which we had demonstrated that M1 macrophages promoted T2D VC. In vivo studies found that pravastatin significantly upregulated TMCA levels in the feces and serum of T2D mice transplanted with *BF* and promoted the macrophage M1 polarization in bone marrow and the osteoblastic differentiation of aortic cells. Similar results were obtained in T2D mice after intravenous infusion of TMCA.

**Conclusions:**

Promoting intestinal *BF* to secrete TMCA, which leads to macrophage M1 polarization, is an important mechanism by which pravastatin promotes calcification, and the result will be used for the optimization of clinical medication strategies of pravastatin supplying a theoretical basis and experimental basis.

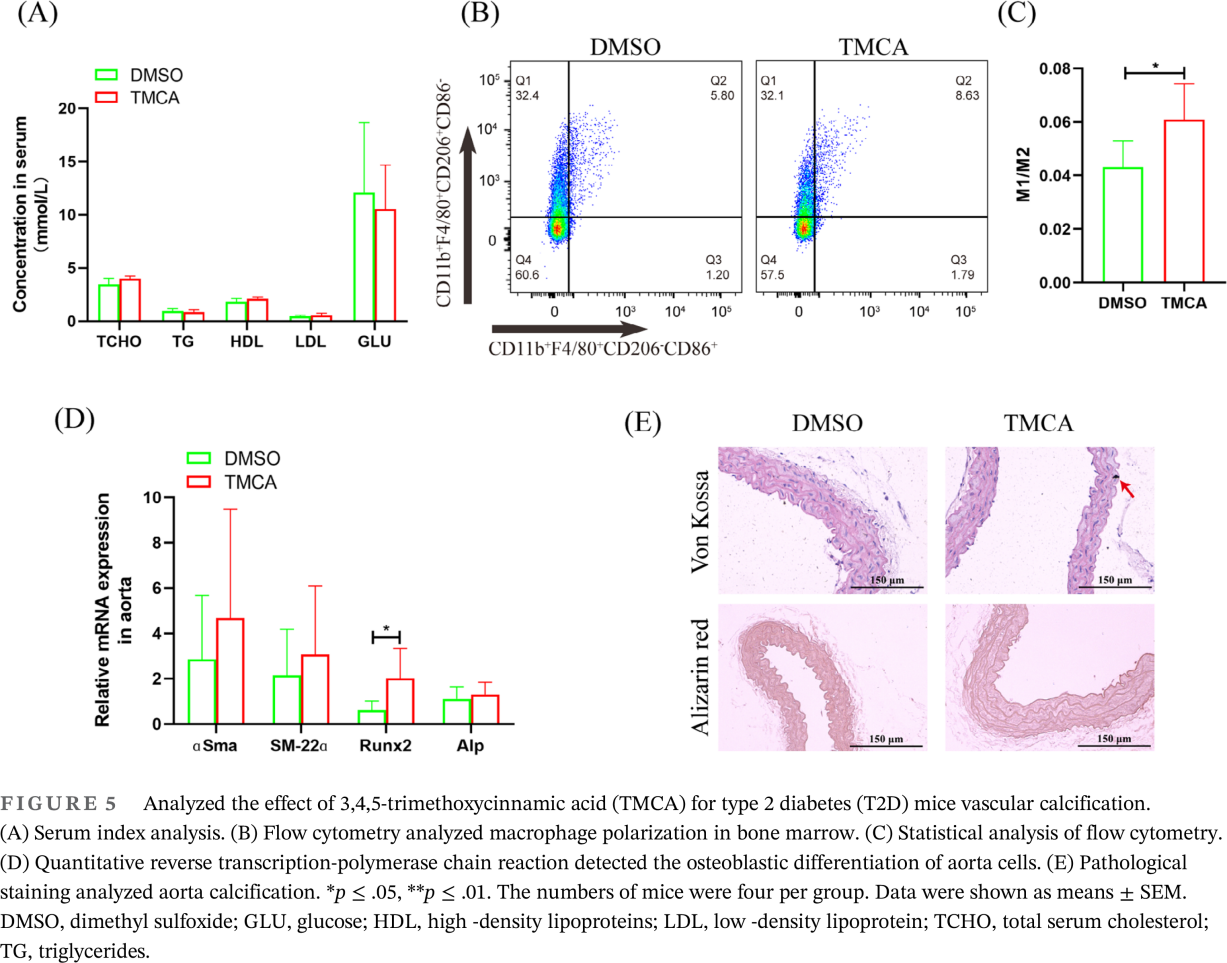

## Graphical abstract

Highlights

- It is first reported that pravastatin treatment induce *Bacteroides fragilis* activation.
- It is first reported that 3,4,5‐trimethoxycinnamic acid (TMCA) is an important metabolite of *Bacteroides fragilis* after pravastatin treatment
- It is first reported that TMCA promotes type 2 diabetes vascular calcification via inducing macrophage M1 polarization.

## 1 INTRODUCTION

Diabetes is a metabolic disease with disordered glucose metabolism, which can easily lead to atherosclerosis, vascular calcification and other life‐threatening cardiovascular diseases.^1^, ^2^, ^3^ Intestinal microbes are closely related to the metabolism of host substances. In the pathological process, intestinal microbes produce many kinds of bioactive metabolites, which trigger the inflammatory response,^4^, ^5^ and affect the process of cardiovascular disease.^6^, ^7^

Recently, it has been found that statins, a common oral lipid‐lowering drug for diabetes patients (more than 80% is excreted through feces), are prone to induce vascular calcification (VC),^8^, ^9^ but the mechanism by which statins induce type 2 diabetes (T2D) VC is not clear. Studies have found that statin therapy may increase the abundance of *Bacteroides*,^10^, ^11^ and *Bacteroides fragilis* (*BF*) can increase the effect of metformin in regulating blood glucose.^12^, ^13^ Therefore, does *BF* mediate the effect of statins on diabetic vascular calcification?

Metabolites are important ways by which intestinal bacteria affect the host. For example, the increase in trimethylamine oxide in peripheral blood is positively correlated with the occurrence and development of cardiovascular disease^14^; inhibition of trimethylamine oxide production effectively reduces the formation of macrophage foam cells and the development of atherosclerosis.^15^, ^16^ These results showed that intestinal bacteria secrete metabolites to regulate the inflammatory response, which may be an important way for intestinal bacteria to participate in VC.

VC is a chronic inflammatory disease that is closely related to the abnormal activation of macrophages, B cells, T cells, and other immune cells.^17^ We previously confirmed that peripheral macrophages in T2D patients are prone to polarize into the M1 phenotype and secrete inflammatory factors and extracellular vesicles to promote VC.^18^, ^19^, ^20^ Are macrophages also the key target cells of *BF* to promote T2D VC?

Therefore, this research analyzes the effect of pravastatin on intestinal *BF* at the clinical, animal, and cell levels and illustrates the mechanism of the main metabolites secreted by *BF* to promote T2D VC. The results will provide a theoretical and experimental basis for the optimization of the clinical use of pravastatin.

## 2 MATERIALS AND METHODS

This research was approved by the Animal Welfare and Research Ethics Committee of the Institute of University of South China and was approved by the Ethics Committee of the First Affiliated Hospital of University of South China.

### 2.1 Cell culture

RAW264.7 was purchased from the National Infrastructure of Cell Line Resource (China Center for Type Culture Collection) and cultured in high‐ or low‐glucose Dulbecco's modified Eagle's medium (DMEM, Gibco BRL, Grand Island, USA) with 10% fetal bovine serum (FBS, Gibco, Australia) and 100 U/mL penicillin–streptomycin at 37°C and 5% CO_2_.

### 2.2 Intestinal bacteria detection

Seven patients (male/female = 5/2, average age of 54.9 ± 10.4), who were diagnosed as T2D post admission and needed statin therapy in clinic, were selected in this research. The patients were without digestive tract diseases and liver diseases. Fecal samples were collected before taking pravastatin, and the second fecal sample was collected 3 days after the patient took 20 mg pravastatin (QN oral medicine). The fecal samples were used for 16S rRNA sequencing. Besides, the feces DNA were exacted according the instruction of Stool Genomic DNA Extraction Kit (Solarbio). *BF* was detected through polymerase chain reaction (PCR) assay. The primers were showed in Table 1. The reaction program was as follows: 95°C for 2 min; 40 cycles at 95°C for 15 s, and 60°C for 30 s. The experiment was performed in a LightCycler 480 II. In addition, parts of the patients’ feces were used to detect intestinal bacteria through 16S rRNA sequencing at Genesky Bio‐Tech Co., Ltd.

**TABLE 1:** Primers sequence.

Gene name	Primer sequence (5′ → 3′)
B. fragilis 16S‐F^a^	TGGACTGCAACTGACACTGA
B. fragilis 16S‐R^a^	GCCGCTTACTGTATATCGCA
Runx2‐F	TGTCCGCCACCACTCACTACC
Runx2‐R	GGGAACTGATAGGATGCTGACGAAG
Alp‐F	CACGGCGTCCATGAGCAGAAC
Alp‐R	CAGGCACAGTGGTCAAGGTTGG
SM22ɑ‐F	ACTCTAATGGCTTTGGGCAGTTTGG
SM22ɑ‐R	CCTCTTATGCTCCTGGGCTTTCTTC
αSma‐F	CGTGGCTATTCCTTCGTGACTACTG
αSma‐R	CGTCAGGCAGTTCGTAGCTCTTC
Tnfɑ‐F	GCGACGTGGAACTGGCAGAAG
Tnfɑ‐R	GCCACAAGCAGGAATGAGAAGAGG
Il‐1β‐F	CACTACAGGCTCCGAGATGAACAAC
Il‐1β‐R	TGTCGTTGCTTGGTTCTCCTTGTAC
Gapdh‐F	TGTTTCCTCGTCCCGTAG
Gapdh‐R	CAATCTCCACTTTGCCACT

*Note*: Detection of enterotoxigenic *Bacteroides fragilis* in patients with ulcerative colitis.

^a^ The primers were referenced in the Zamani et al. report.

### 2.3 Flow cytometry

RAW264.7 cells were divided into three groups (50 μL/sample): blank group, isotype control group, and experimental group. The isotype control group received 1 μL Rat IgG2a Kappa‐FITC, 1 μL Rat IgG2b Kappa‐PerCP‐Cy5.5, and 1 μL Rat IgG2a Kappa‐APC. The experimental group received 1 μL CD86 monoclonal antibody‐FITC, 1 μL CD11b monoclonal antibody‐PerCP‐Cy5.5, and 1 μL F4/80 monoclonal antibody‐APC. All groups were incubated at room temperature for 30 min. If the sample was peripheral blood, a treatment step was added for incubation with 1 mL red blood cell lysate (BD Biosciences) for 10 min. Then, the samples were resuspended in 1 mL phosphate‐buffered saline (PBS) and concentrated at 250 g for 5 min. Finally, the pellet was resuspended in 300 μL PBS and detected using BD FACS AriaTM II. All flow cytometry antibodies were purchased from Thermo Fisher Scientific Inc. The antibodies information were showed in Table 2.

**TABLE 2:** Antibodies used in the experiments.

Antibody name	Source	Manufacturer	Catalog # (RRID)	Application
Anti‐F4/80‐APC	Rat	Thermo Fisher Scientific	#AB_2784647	FC (1:50)
Anti‐CD11b‐Percp‐Cy5.5	Rat	Thermo Fisher Scientific	45–0112‐80#AB_953560	FC (1:50)
Anti‐CD86‐FITC	Rat	BD Pharmingen	553 691#AB_394993	FC (1:50)
Anti‐CD206‐PE	Rat	Thermo Fisher Scientific	12–2061‐821#AB_2637421	FC (1:50)
ɑ‐SMA	Rabbit	Cell signaling	19 245#AB_2734735	WB (1:1000)
SM‐22ɑ	Rabbit	Abcam	40 471#AB_443021	WB (1:1000)
Runx2	Rabbit	Abcam	20 700‐1‐AP#AB_2722783	WB (1:1000)
				IHC (1:100)
HRP‐conjugated GAPDH	Mouse	Proteintech	10 494‐1‐AP#AB_2737588	WB (1:3000)

Abbreviations: FC: Flow cytometry; HRP, horseradish peroxidase; IHC, immunohistochemistry; WB, Western blotting.

### 2.4 RNA extraction and cDNA synthesis

Total RNA from cells or tissues was extracted using the RNA simple Total RNA Kit (TianGen Biotech (Beijing) Co., Ltd., China) or the miRNA Purification Kit (CWBIO, Beijing, China). Total cDNA was synthesized using the Revert Aid First Strand cDNA Synthesis Kit (Thermo Fisher Scientific Inc., Waltham, MA USA), and miRNA was synthesized using the Mir‐XTM miRNA First Strand Synthesis Kit (Takara Biomedical Technology (Beijing) Co., Ltd. Beijing, China).

### 2.5 Quantitative Reverse‐Transcription PCR

The 20 μL reaction volume of quantitative reverse‐transcription (qRT)‐PCR contained 10 μL 2 × SYBR Green PCR Mastermix (Takara), 1 μL forward primer, and 1 μL reverse primer (Table 1), 1 μL cDNA template, and 7 μL ddH_2_O. The reaction program was as follows: 95°C for 2 min; 40 cycles at 95°C for 15 s, and 60°C for 30 s. The experiment was performed in a LightCycler 480 II.

### 2.6 Cellular protein extraction

Cellular protein was extracted using the kit of Cell Lysis Buffer for Western and IP (Beyotime Biotechnology, Shanghai, China). The protein concentration was determined using a BCA Protein Assay Kit (Solarbio).

### 2.7 Preparation of mouse anti‐*ArsR* antibody

*ArsR* sequence, for which the 5′‐end coding regions had HRV‐3C restriction site, was inserted into pet‐32a (+) plasmid. The recombinant plasmid was transferred into *Escherichia coli* BL21 bacteria and then plating, picking bacteria, activation, expansion, and IPTG induction. The bacterial protein was extracted, purified by magnetic beads, HRV‐3C enzyme digestion, and protein concentration. The protein was collected and stored at −80°C.

Then, 100 μg of purified protein was added into Freund's complete adjuvant (Sigma Chemical, USA), fully mixed to chylous state, and administered by abdominal subcutaneous injection to C57BL/6J mouse (6 points, each point 0.1 mL). Two weeks later, 50 μg of purified protein was taken and fully mixed with Freund's incomplete adjuvant (Sigma Chemical, USA) to chylous state, injected to mouse as previous description, once a week for 2 weeks. The *ArsR* antibody titer was detected by Dot‐ELISA, the mouse was anesthetized with isoflurane, and orbital blood was collected and kept at room temperature for 1 h. Next, the blood was centrifuged at 4°C with 2500 g for 30 min, and the serum was collection and storage in −80°C.

### 2.8 Preparation of *BF* competence and metabolome sequencing

*BF* single colony was picked from LB solid plate and was anaerobically cultured in LB liquid medium at 37°C until OD (optical density)_600nm_ >1.0. Then, the bacterial solution was transferred to 100 mL fresh LB culture medium (1:50) and was anaerobically cultured at 37°C until OD_600nm_ >0.6. *BF* was collected by centrifuged at 4°C with 1980 g for 10 min and resuspended with 25 mL pre‐cool Inoue solution (1.088 g MnCl_2_.4H_2_O, 0.22 g CaCl_2_∙2H_2_O, 1.865 g KCl, 2 mL PIPES solution [0.5 M, pH 6.7], ddH_2_O to 100 mL). After being centrifuged at 4°C with 1980 g for 10 min, BF was resuspended in appropriate pre‐cooled Inoue solution which contained 7.5% dimethyl sulfoxide (DMSO). After being placed on ice for 10 min, it was subpacked and stored at −80°C.

The pet32a‐*ArsR* and control plasmid were respectively added to *BF* competent bacteria, followed with ice bath 25 min, 42°C heat shock 90 s, and ice bath 2 min. A 700ul LB liquid medium was added to the bacteria, and then 37°C anaerobic cultures 1 h. After centrifuged at 2500 g for 1 min, the precipitate was respectively coated on a LB solid plate containing ampicillin and anaerobically cultured overnight at 37°C. The single colonies were selected for expansion culture. After PCR detection, the bacteria were collected and sent to GENESKY (Genesky Biotechnologies Inc., Shanghai, China) for metabolomics sequencing.

### 2.9 Sodium dodecyl‐sulfate polyacrylamide gel electrophoresis (SDS‐PAGE) and mass spectrometry

Protein samples were separated by SDS‐PAGE, and an 8% separation gel was prepared and electrophoresed at 80 V for 20 min, followed by 120 V for 90 min. Then, the gel was stained with Coomassie G‐250. The protein was excised from the gels and identified by mass spectrometry at Shanghai iproteome Biotechnology Co., Ltd.

### 2.10 Western blotting

Protein samples were separated by SDS‐PAGE. Then, the protein was transferred onto a polyvinylidene fluoride membrane using a semi‐dry transfer apparatus (Bio‐Rad Laboratories, Inc., CA, USA) according to the manufacturer's instructions. The membrane was then blocked at room temperature for 1 h in blocking buffer (tris‐buffered saline containing 5% nonfat powdered milk and 0.1% Tween‐20), and then incubated with primary antibody overnight at 4°C. After the membrane was washed three times for 5 min each, it was incubated with secondary antibody linked horseradish peroxidase at room temperature for 40 min and washed two times for 20 min each. Finally, the membrane was developed using an Immobilon Western Chemiluminescent HRP Substrate kit (EMD Millipore Corporation, MA, USA) and analyzed using ChemiDoc™ XRS+ (Bio‐Rad). The antibodies information were shown in Table 2.

### 2.11 Generation of T2D mice

T2D mice were generated as previously reported.^19^, ^20^ Mice were group housed (2–5 mice per cage) at a controlled temperature (22 ± 2°C) under a 12‐h light/dark cycle with free access to food and water. The mice adapted to their new environment for 7 days before the behavioral tests. All animals were used for only one procedure. Simply, 8‐ to 10‐week‐old mice (C57/BL6 background) were fed a chow fat diet or a high‐fat diet with 10% (TP23102) or 45% (TP 23100) of the energy from fat, respectively (Trophic Animal Feed High‐tech Co., Ltd., Jiangsu, China). A month later, streptozotocin (Sigma Chemical, USA) was intraperitoneally injected at 25 mg/kg for 3 consecutive days. Serum insulin was detected using a Mouse INS ELISA Kit (Feiya Biotechnology Co., Ltd., Jiang Su, China). The homeostasis model assessment‐insulin resistance (HOMA‐IR) index was calculated according to the formula HOMA‐IR = Fasting blood glucose (mg/dL) × fasting serum insulin (mU/mL)/405.

### 2.12 Bone marrow‐derived macrophages (BMDM) preparation

Bone marrow cells were obtained from the tibias and femurs of 6‐ to 8‐week‐old mice. After isoflurane anesthesia, mononuclear cells were isolated with Mouse Bone Marrow Mononuclear Cell Separation Kit (Solarbio) and cultured in complete DMEM containing 10% heat‐inactivated FBS (Gibco), 0.5% penicillin–streptomycin, 10 ng/mL granulocyte‐macrophage colony‐stimulating factor (Beijing Solarbio Science & Technology Co., Ltd., China) and 5 ng/mL macrophage colony stimulating factor (Solarbio). After culturing at 37°C and 5% CO_2_ overnight, the nonadherent or weakly adherent cells were transferred into another 10 cm plastic dish. The culture medium was changed after 2 days and 5 days. Finally, adherent BMDM were collected for further analysis.

### 2.13 *BF* translation and pravastatin treatment

Antibiotic cocktails (neomycin 1 mg/mL, streptomycin 1 mg/mL, and bacitracin 1 mg/mL) were added to mice drinking water for 3 days. The *BF* cultured in vitro, which was calculated by OD_600nm_‐bacterial number curve, diluted with PBS to 5 × 10^8^ CFU/mL. All of the mice were intragastrically administered with 0.1 mL bacterial suspension per mouse. During *BF* transplantation, 0.15 mg/mL pravastatin or equal volume DMSO was added into drinking water, which the concentration was calculated according human dose 20 mg/day (mouse equivalent conversion coefficient was 12.3) and the average daily drinking water was about 11 mL per mouse.

### 2.14 TMCA injection

The 3,4,5‐trimethoxycinnamic acid (TMCA) was 39 ng/mL in the serum of T2D mouse and 647 ng/mL on average in the the serum of T2D mice treated with pravastatin and *BF*. The total blood volume was approximately 1.8 mL. Therefore, the mice were injected with 1094.4 ng TMCA or equal volume DMSO via the caudate vein two times per week.

### 2.15 Histology analysis

Five randomly selected mice per group were subjected to deep anesthesia using 10% chloral hydrate (3.5 mL/kg, i.p.) and the aorta was extracted and fixed in 4% paraformaldehyde. Fixed sample of alveolar lavage fluid was embedded in paraffin and sectioned at 5 μm, followed by hematoxylin and eosin, Alizarin red, and von Kossa, and observed using the EVOS M7000 (Invitrogen, Carlsbad, CA). All of the detection was performed according the introduction of kits, which were all purchased from Solarbio (Solarbio Science and Technology Co. Ltd., Bei Jing, China).

### 2.16 Immunohistochemistry analysis

Deparaffinated sections of the aorta were prepared by heating the sample for 20 min, cooling the sample at room temperature, and then washing the sample three times with 0.01 M PBS (pH 7.2–7.6) for 3 min. Endogenous peroxidase activity was blocked by incubating the sections in 1.0% periodic acid for 10 min and then washing them three times with 0.01 M PBS for 3 min. The sections were incubated with rabbit anti‐mouse Runx2 (ab76956, abcam) at 4°C overnight. After the sections were washed three times with PBS for 5 min, they were incubated with anti‐rabbit IgG‐HRP at 37°C for 30 min. They were then washed three times with PBS for 5 min and further treated with Metal Enhanced DAB Substrate Kit (Solarbio) at room temperature for 1–5 min. Following hematoxylin staining, alcoholic dehydration, xylene treatment and application of a neutral resin sealing sheet, the sections were visualized using a DAB kit (ZSGB‐BIO). The antibodies information were showed in Table 2.

### 2.17 Index detection of serum

After the mice in deep anesthesia, blood was obtained through the enucleated eyeball and then incubated at 37°C for 10 min. The samples were centrifugalized at 4000 rpm for 10 min, and serum was collected. Then the index in the serum was detected by Roche cobas 8000 automatic biochemical analyzers.

### 2.18 ELISA detection

TMCA concentration was detected according the instruction of TMCA ELISA Kit (Feiya Biotechnology Co., Ltd., Jiang Su, China). Tumor necrosis factor alpha (TNFɑ) concentration was detected according to the instruction in the Mouse TNFA/TNF Alpha ELISA Kit (Boster Biological Technology co.Itd, Wu Han, China).

### 2.19 Bioinformatics analysis

We downloaded the transcriptome data about *BF* in response to environmental changes from the Gene Expression Omnibus (GEO) database (https://www.ncbi.nlm.nih.gov/gds/?term=). The two transcriptome data sets GSE4583 and GSE129572. GSE4583 were used to analyze the effect of different oxygen concentration (anaerobic culture, 5% oxygen, oxygen stressed) for gene expression of *BF*. GSE129572 analyzed the effect of different types sugar (glucose or bovine alpha 1 acid glycoprotein) for gene expression of *BF*.

### 2.20 Statistical analysis

All experiments were repeated at least three times. The data were analyzed using GraphPad Prism 7.00 software, and the results are shown as the mean ± SEM. Student's *t* test or one‐way analysis of variance with Bonferroni correction was used to assess statistical significance. *p* < .05 or *p* < .01 were considered significant or very significant, respectively.

## 3 RESULTS

### 3.1 Pravastatin significantly promotes feces *BF* proliferation in T2D patients

**FIGURE 1:**
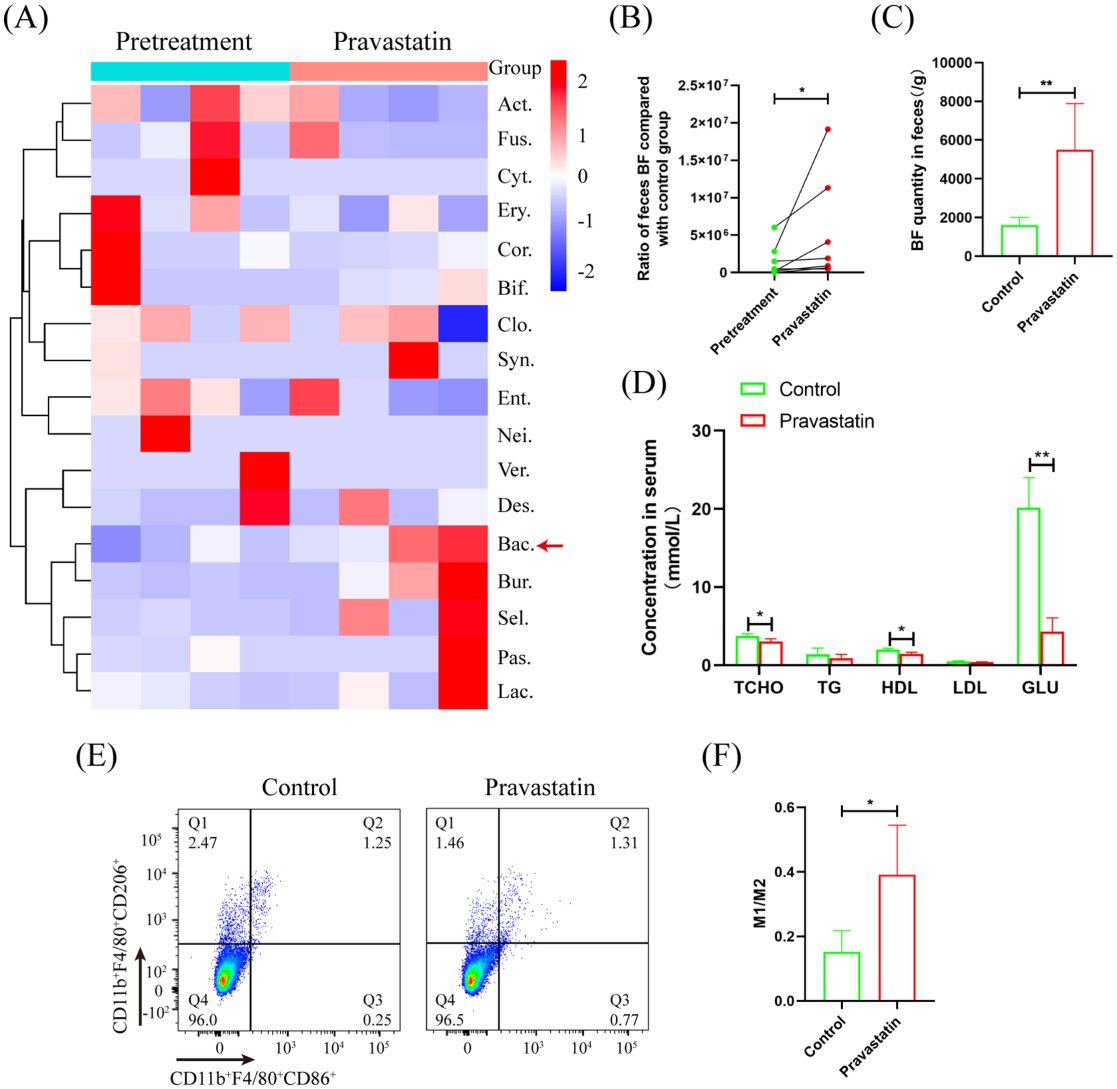
Analyzed the effect of pravastatin for feces *Bacteroides fragilis* (*BF*) and trimethoxycinnamic acid (TMCA) in type 2 diabetes (T2D) patients. (A) 16S rRNA sequencing analyzed *BF* in T2D patients feces *n* = 4. (B) Quantitative reverse transcription‐polymerase chain reaction (qRT‐PCR) detected the quantity of *BF* in T2D patients feces, *n* = 7. (C) qRT‐PCR detected the quantity of *BF* in T2D mice feces after pravastatin treated. (D) Serum index analysis. (E) Flow cytometry analyzed the effect of pravastatin for macrophage polarization in bone marrow. (F) Statistical analysis of flow cytometry. *p ≤ .05, **p ≤ .01. The numbers of mice were four per group. Data were shown as means ± SEM. Act, *Actinomycetales*; Bac, *Bacteroidales*; Bif, *Bifidobacteriales*; Bur, *Burkholderiales*; Cor, *Coriobacteriales*; Clo, *Clostridiales*; Cyt, *Cytophagales*; Des, *Desulfovibrionales*; Ent, *Enterobacteriales*; Ery, *Erysipelotrichales*; Fus, *Fusobacteriales*; GLU, glucose; HDL, high‐density lipoproteins; Lac, *Lactobacillales*; LDL, low‐density lipoprotein; Nei, *Neisseriales*; Sel, *Selenomonadales*; Syn, *Synergistales*; Pas, *Pasteurellales*; TCHO, total serum cholesterol; TG, triglycerides; Ver, *Verrucomicrobiales*.

After pravastatin treatment for 3 days, 16S rRNA sequencing showed that the *Bacteroides* order increased significantly in the feces of the seven patients (Figure 1A). qRT‐PCR showed that *BF* per gram of feces increased significantly to 3.42‐fold after taking pravastatin (Figure 1B). In addition, after T2D mice were treated with pravastatin, *BF* was significantly increased to 3.4‐fold (Figure 1C). Biochemical tests showed that serum total cholesterol (TCHO), high‐density lipoprotein (HDL), and glucose (GLU) were significantly downregulated to 1.23‐, 1.38‐, and 4.69‐fold, respectively (Figure 1D). Flow cytometry found that macrophages in bone marrow were significantly polarized to the M1 phenotype (Figure 1E,F).

### 3.2 Pravastatin promotes TMCA secretion in *BF*

**FIGURE 2:**
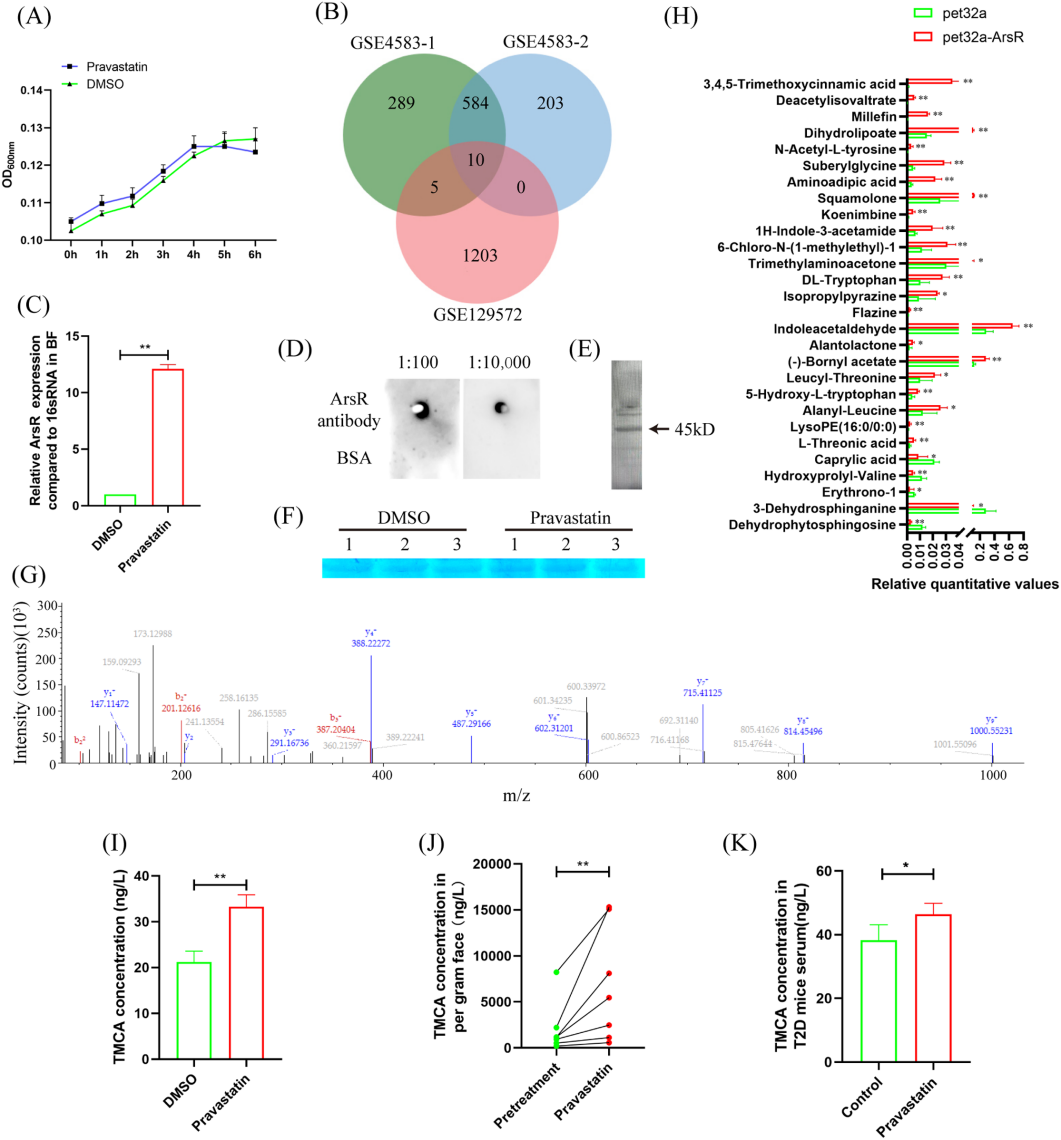
Analyzed the effect of pravastatin for *Bacteroides fragilis* (*BF*) activation. (A) Detected the effect of pravastatin for *BF* proliferation. (B) Analyzed the key gene of *BF* coping with environmental change. (C) quantitative reverse transcription‐polymerase chain reaction (qRT‐PCR) detected *ArsR* expression after pravastatin treated in *BF*. (D) Dot‐ELISA analyzed the antibody titer of mouse anti‐*ArsR*. (E) Western blot detected *ArsR* expression in *BF*. (F) Sodium dodecyl‐sulfate polyacrylamide gel electrophoresis (SDS‐PAGE) analyzed *ArsR* expression after *BF* treated with pravastatin. (G) Mass spectrometry detected *ArsR* antibody‐combined protein. (H) Metabolomics analyzed the divergent metabolites after *BF* overexpressed *ArsR*. (I) ELISA analyzed trimethoxycinnamic acid (TMCA) concentration in culture supernatant of *BF* after pravastatin treated. (J) ELISA detected TMCA concentration in T2D patients' feces. (K) ELISA detected serum TMCA concentration in T2D mice after pravastatin treated. *p ≤ .05, **p ≤ .01. Each experiment was repeated more than three times (*n* ≥ 3). were was shown as means ± SEM. BSA, bovine serum albumin; DMSO, dimethyl sulfoxide; OD, optical density; T2D, type 2 diabetes.

In vitro, 10 μg/mL pravastatin had no significant effect on *BF* proliferation (Figure 2A). In the GSE4583 and GSE129572 data sets, the genes with change fold changes >1.5 and with significant changes were used for Venn diagram analysis, and 10 genes were found as follows: hypothetical protein, *ArsR* family transcriptional regulator, formate‐tetrahydrofolate ligase, glutamine amidotransferase, L‐fucose isomerase, ATP synthase gamma chain, 30S ribosomal protein S20, 50S ribosomal protein L34, 8‐amino‐7‐oxononanoate synthase, and CTP synthase (Figure 2B). Among these genes, *ArsR*, which was associated with bacterial resistance to environmental mutations, was significantly increased to 12.11‐fold after *BF* treatment with pravastatin (Figure 2C). Western blot and SDS‐PAGE also verified that pravastatin promoted *ArsR* expression in the *BF* (Figure 2D–G). To clarify the effect of *ArsR* upregulated expression on *BF* metabolism, the pet32a‐*ArsR* plasmid was transferred into *BF*. Metabolome sequencing analysis showed that TMCA was increased more than the other metabolites, and it was significantly to 60.18‐fold (Figure 2H). After *BF* was treated with pravastatin, TMCA in the supernatant was also significantly upregulated by 1.57‐fold (Figure 2I). Importantly, after pravastatin treatment, the concentration of TMCA significantly increased to 3.33‐fold in the patient's feces (Figure 2J), and significantly increased to 1.21‐fold in the T2D mouse serum (Figure 2K).

### 3.3 TMCA promotes vascular smooth muscle cell osteogenic differentiation by inducing macrophage M1 polarization

**FIGURE 3:**
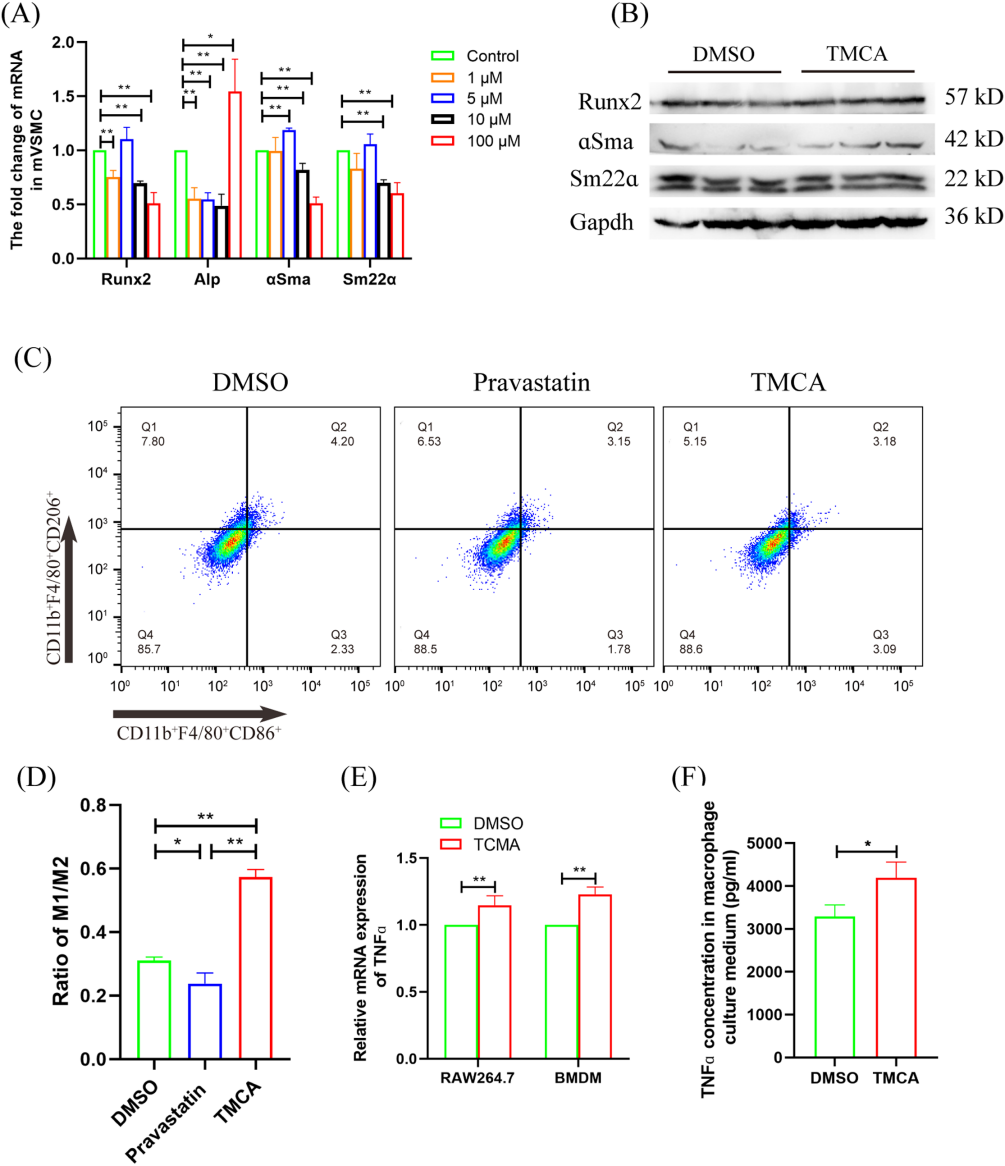
Analyzed the mechanism of trimethoxycinnamic acid (TMCA) promoted vascular calcification. (A) Quantitative reverse transcription‐polymerase chain reaction (qRT‐PCR) analyzed the effect of different TMCA concentration for vascular smooth muscle cell (VSMC) osteoblastic differentiation. (B) Western blot analyzed the effect of TMCA for VSMC osteoblastic differentiation. (C) Flow cytometry analyzed the effect of TMCA or pravastatin for macrophage polarization. (D) Statistical analysis of flow cytometry. (E) qRT‐PCR analyzed the expression of inflammatory factors after RAW264.7 or BMDM treated with TMCA. (F) ELISA analyzed TNFɑ concentration after macrophage treated with TMCA. *p ≤ .05, **p ≤ .01. Each experiment was repeated more than three times (*n* ≥ 3). Data were shown as means ± SEM. BMDM, bone marrow‐derived macrophages; DMSO, dimethyl sulfoxide; TNFɑ, tumor necrosis factor alpha.

As an important metabolite secreted by *BF*, we stimulated mouse vascular smooth muscle cell (VSMC) with different concentrations of TMCA. The results showed that although the smooth muscle marker genes (*ɑSma* and *Sm22ɑ*) decreased, *Runx2*, which was the osteogenic differentiation initiation gene, did not increase significantly, and the calcification marker gene *Alp* did not increase significantly (except for the highest concentration of 100 μM TMCA) (Figure 3A). Western blot also found that there was no significant change in VSMC osteogenic marker genes and smooth muscle marker genes after 5 μM TMCA treatment (Figure 3B). Our group previously confirmed that one of the causes of T2D VC is the immune response mediated by macrophage M1 polarization. Therefore, we stimulated RAW264.7 cells with 5 μM TMCA or pravastatin and found that TMCA significantly promoted macrophage M1 polarization, up to 1.84 times; pravastatin significantly inhibited macrophage M1 polarization, down to 0.76 times (Figure 3C,D). Furthermore, qRT‐PCR analysis showed that 5 μM TMCA significantly upregulated the expression of TNFɑ in RAW264.7 cells or BMDMs by 1.15‐fold and 1.23‐fold, respectively (Figure 3E). ELISA assay also found that TNFɑ in the supernatant of RAW264.7 cells was significantly upregulated by 1.27‐fold after TMCA treatment (Figure 3F).

### 3.4 Promotion of TMCA secretion in *BF* is an important mechanism by which pravastatin promotes T2D VC

**FIGURE 4:**
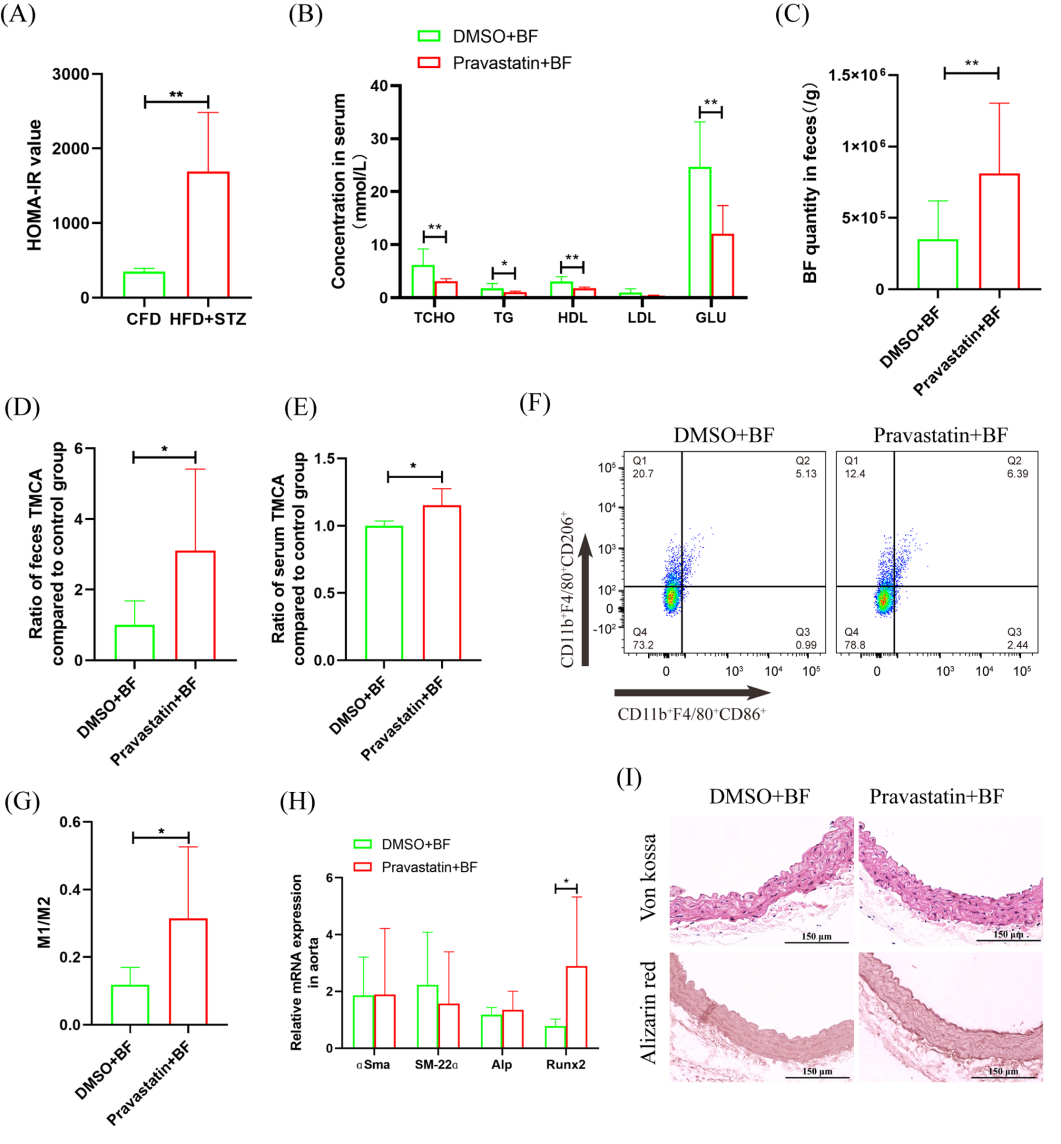
Analyzed the effect of pravastatin and *Bacteroides fragilis* (*BF*) treatment for type 2 diabetes (T2D) mice vascular calcification. (A) Homeostasis model assessment‐insulin resistance (HOMA‐IR) index analysis. (B) Serum index analysis. (C) Quantitative reverse transcription‐polymerase chain reaction (qRT‐PCR) detected the *BF* in feces. (D) ELISA detected trimethoxycinnamic acid (TMCA) concentration in feces. (E) ELISA detected TMCA concentration in serum. (F) Flow cytometry analyzed macrophage polarization in bone marrow. (G) Statistical analysis of flow cytometry. (H) qRT‐PCR detected the osteoblastic differentiation of aorta cells. (I) Pathological staining analyzed aorta calcification. *p ≤ .05, **p ≤ .01. The numbers of mice in DMSO + *BF* or pravastatin + *BF* groups were five or six, respectively. Data were shown as means ± SEM. CFD, chow fat diet; DMSO, dimethyl sulfoxide; GLU, glucose; HDL, high ‐density lipoproteins; HFD, high‐fat diet; LDL, low ‐density lipoprotein; STZ, streptozotocin; TCHO, total serum cholesterol; TG, triglycerides.

Furthermore, T2D mice were constructed, which had obvious insulin resistance, were constructed (Figure 4A). After antibiotic cocktail treatment, *BF* transplantation and pravastatin treatment were performed for 2 weeks. Biochemical tests showed that TCHO, triglyceride (TG), HDL, and GLU were significantly downregulated to 0.50‐, 0.56‐, 0.60‐, and 2.05‐fold, respectively. Low‐density lipoprotein (LDL) was not significantly different, but it was also downregulated to 0.41‐fold (Figure 4B). The qRT‐PCR results showed that *BF* in feces was significantly increased to 2.32‐fold after pravastatin treatment (Figure 4C). ELISA showed that TMCA in feces and serum was significantly increased to 3.10‐fold and 1.15‐fold, respectively (Figure 4D,E). Flow cytometry analysis showed that M1 macrophages in bone marrow were significantly increased to 1.63‐fold, and M2 macrophages were significantly downregulated to 0.70‐fold (Figure 4F,G). Aortic calcification detection showed that pravastatin treatment had no significant effect on aortic calcium deposition and calcification (Figure 4I) but significantly promoted the expression of the osteogenic differentiation promoter *Runx2*, which was significantly increased to 3.69‐fold (Figure 4H).

### 3.5 TMCA promotes T2D VC

**FIGURE 5:**
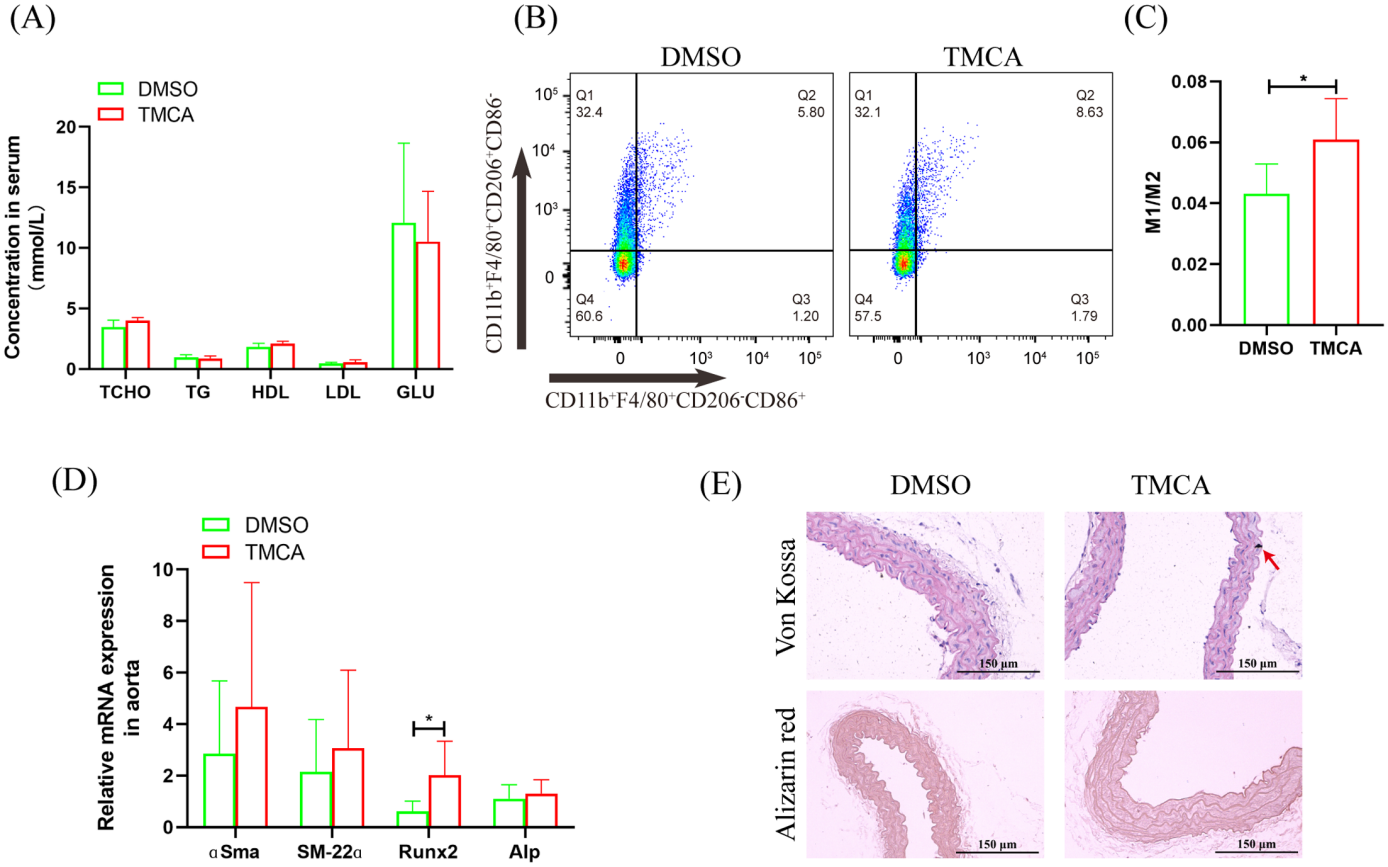
Analyzed the effect of 3,4,5‐trimethoxycinnamic acid (TMCA) for type 2 diabetes (T2D) mice vascular calcification. (A) Serum index analysis. (B) Flow cytometry analyzed macrophage polarization in bone marrow. (C) Statistical analysis of flow cytometry. (D) Quantitative reverse transcription‐polymerase chain reaction detected the osteoblastic differentiation of aorta cells. (E) Pathological staining analyzed aorta calcification. *p ≤ .05, **p ≤ .01. The numbers of mice were four per group. Data were shown as means ± SEM. DMSO, dimethyl sulfoxide; GLU, glucose; HDL, high ‐density lipoproteins; LDL, low ‐density lipoprotein; TCHO, total serum cholesterol; TG, triglycerides.

After TMCA treated 2 weeks, TCHO, TG, HDL, LDL, and GLU had no significant change (Figure 5A). Flow cytometry analysis showed that macrophages polarized to an inflammation phenotype in the bone marrow (Figure 5B,C). qRT‐PCR showed that *Runx2* mRNA was significantly increased in the aorta (Figure 5D). Importantly, von Kossa staining revealed aortic calcium deposition after TMCA treatment, although no calcification was found by alizarin red staining (Figure 5E).

## 4 DISCUSSION

Patients with diabetes are prone to VC, which increases the incidence of cardiovascular adverse events.^21^ As an oral lipid‐lowering drug commonly used in diabetes, statins are positively correlated with the occurrence of VC.^22^ However, the mechanism by which statins promote vascular calcification is unclear, leading to the adverse effects for the optimization of clinical use strategies of statins.

Intestinal is an important absorption site of oral drugs, and intestinal bacteria play an important role in drug metabolism and efficacy.^23^ Studies have found that intestinal *BF* helps to improve the hypoglycemic effect of metformin.^12^ Therefore, a retrospective clinical study was carried out, and the results showed that diabetic patients treated with pravastatin had significantly increased fecal *BF* (Figure 1A,B). The results were also verified in animal model (Figure 1C). Intestinal bacteria affect the host mainly through two aspects: changes in proliferative activity and changes in functional metabolites.^24^ In vitro experiments showed that pravastatin had little effect on the proliferation of *BF* (Figure 2A), but it could significantly promote the proliferation of *BF* in vivo (Figure 4C). These inconsistent results may be due to the richer nutrition in the in vivo environment. These results suggest that *BF* may be an important intestinal bacteria associated with pravastatin treatment.

The change in genes for intestinal bacteria tackling the environment is an important reason for metabolite alterations.^25^, ^26^ Based on the GSE4583 and GSE129572 data sets in the GEO database, we found 10 potential genes in *BF* that changed significantly with the environment (Figure 2B). Among these genes, *ArsR* is classified as a member of the DNA‐binding transcription inhibitory protein family, which is common in intestinal G^+^ and G^−^ bacteria^27^ and is involved in various important cellular events of bacteria (such as primary and secondary metabolism).^28^ This study found that the *ArsR* gene was significantly upregulated after pravastatin treatment (Figure 2C–G), whereas pravastatin treatment or *ArsR* overexpression all significantly promoted the upregulation of the metabolite TMCA in the *BF* (Figure 2H,I). Importantly, after patients were treated with pravastatin, TMCA in feces also increased significantly (Figure 2J). The results show that TMCA is an important metabolite of pravastatin that promotes *BF* secretion. Clarifying the role of TMCA in the process of VC is helpful for elucidating the molecular mechanism by which pravastatin promotes T2D VC.

TMCA esters and amides are special structural scaffolds in drug discovery and are widely distributed in natural products.^29^ Therefore, they produce a variety of pharmacological functions related to treatment, such as antianxiety,^30^ antiepilepsy,^31^ and antiarrhythmia,^32^ and so on. However, there are no reports on TMCA and diabetes and vascular calcification. Therefore, we further found that TMCA had a limited effect on the osteogenic differentiation of VSMCs but could significantly promote macrophage M1 polarization (Figure 3, 4, 5). We have previously confirmed that macrophages are prone to M1 polarization in T2D patients and that M1 macrophages secrete extracellular vesicles or inflammatory factors to promote VC.^18^, ^19^, ^20^ These results suggestion that TMCA is an important metabolite of *BF* that promotes T2D VC in patients treated with pravastatin.

In summary, promoting intestinal *BF* to secrete TMCA, which leads to macrophage M1 polarization, is an important mechanism by which pravastatin to promotes calcification. This conclusion will provide a theoretical basis and experimental basis for the optimization of the clinical treatment of patients with diabetes who are taking pravastatin.

## AUTHOR CONTRIBUTIONS

Laboratory experiments, data analysis and manuscript writing were accomplished by Cong Chen; part of experiment were accomplished by Zheng‐Feng Liang, Yu‐Qi He, An‐Qi Li, Yan Gao, and Qun‐Wen Pan; guidance of experimental design and manuscript writing was accomplished by Jing‐Song Cao. All authors have read and approved the final manuscript.

## FUNDING INFORMATION

This work was supported by the Research Foundation of Education Bureau of Hunan Province, China (No. 22A0292); Natural Science Foundation of Hunan Province, China (No. 2021JJ40490 and No. 2021JJ70113).

## CONFLICT OF INTEREST STATEMENT

The authors declare that the research was conducted in the absence of any commercial or financial relationships that could be construed as a potential conflict of interest.

## Data Availability

All data in the article can be requested from the corresponding author.
